# 1,10-Phenanthrolin-1-ium hydrogen (*S*,*S*)-tartrate trihydrate and a correction

**DOI:** 10.1107/S160053680905332X

**Published:** 2009-12-19

**Authors:** Zohreh Derikvand, Marilyn M. Olmstead

**Affiliations:** a Faculty of Science, Department of Chemistry, Islamic Azad University, Khorramabad Branch, Khorramabad, Iran; b Department of Chemistry, University of California, Davis, CA 95616-5292, USA

## Abstract

The title structure, C_12_H_9_N_2_^+^·C_4_H_5_O_6_^−^·3H_2_O, shows that one of the protons of d-tartaric acid has been transferred to 1,10-phenanthroline. The d-hydrogen tartrate anions are joined together in a head-to-tail fashion *via* a short hydrogen bond with donor–acceptor distance of 2.4554 (12) Å, unsymmetrical O—H distances of 1.01 (4) Å and 1.45 (4) Å, and a 174 (4)° O—H—O bond angle. The phenanthrolinium rings are π-stacked with an average separation of 3.58 (11) Å. The structural report corrects a previous report in the literature [Wang *et al.* (2006 ▶). *Acta Cryst.* E**62**, o2508–o2509] of the isostructural l-hydrogen tartrate enanti­omer in which the proton transfer and short hydrogen bond were missed.

## Related literature

For related proton-transfer hydrogen tartrate structures, see: Bai *et al.* (2005 ▶); Paixão *et al.* (1999 ▶); Smith *et al.* (2006 ▶); Su *et al.* (2009 ▶); Suresh *et al.* (2006 ▶); Wang *et al.* (2006 ▶, 2008 ▶); Zhang *et al.* (2006 ▶).
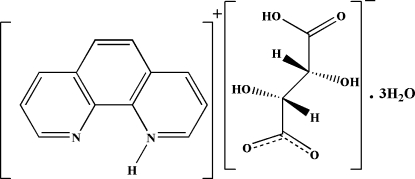


## Experimental

### 

#### Crystal data

C_12_H_9_N_2_^+^·C_4_H_5_O_6_^−^·3H_2_O
*M* *_r_* = 384.34
Orthorhombic, 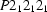

*a* = 7.1163 (14) Å
*b* = 12.482 (3) Å
*c* = 19.466 (4) Å
*V* = 1729.2 (6) Å^3^
*Z* = 4
Mo *K*α radiation
μ = 0.12 mm^−1^
*T* = 90 K
0.42 × 0.21 × 0.13 mm

#### Data collection

Bruker SMART APEXII diffractometer
Absorption correction: multi-scan (*SADABS*; Sheldrick, 1996 ▶) *T*_min_ = 0.880, *T*_max_ = 0.984
39094 measured reflections
3251 independent reflections
3149 reflections with *I* > 2σ(*I*)
*R*_int_ = 0.027

#### Refinement

*R*[*F* ^2^ > 2σ(*F* ^2^)] = 0.032
*wR*(*F* ^2^) = 0.089
*S* = 1.06
3251 reflections
324 parameters
All H-atom parameters refined
Δρ_max_ = 0.41 e Å^−3^
Δρ_min_ = −0.18 e Å^−3^

### 

Data collection: *APEX2* (Bruker, 2007 ▶); cell refinement: *SAINT* (Bruker, 2007 ▶); data reduction: *SAINT*; program(s) used to solve structure: *SHELXTL* (Sheldrick, 2008 ▶); program(s) used to refine structure: *SHELXTL*; molecular graphics: *SHELXTL*; software used to prepare material for publication: *SHELXTL*.

## Supplementary Material

Crystal structure: contains datablocks I, global. DOI: 10.1107/S160053680905332X/nk2019sup1.cif

Structure factors: contains datablocks I. DOI: 10.1107/S160053680905332X/nk2019Isup2.hkl

Additional supplementary materials:  crystallographic information; 3D view; checkCIF report

## Figures and Tables

**Table 1:** Hydrogen-bond geometry (Å, °)

*D*—H⋯*A*	*D*—H	H⋯*A*	*D*⋯*A*	*D*—H⋯*A*
O2—H2*A*⋯O6^i^	1.01 (4)	1.45 (4)	2.4554 (12)	174 (4)
O3—H3*A*⋯O8^ii^	0.86 (2)	1.86 (2)	2.6817 (13)	159 (2)
O4—H4*A*⋯O9^i^	0.81 (3)	1.90 (3)	2.7102 (13)	173 (3)
N2—H2*B*⋯O7	0.912 (19)	1.763 (19)	2.6426 (14)	161.3 (18)
O7—H7*A*⋯O6^iii^	0.91 (2)	1.87 (2)	2.7727 (13)	170 (2)
O7—H7*B*⋯O5	0.81 (3)	2.02 (3)	2.7141 (14)	144 (3)
O7—H7*B*⋯O4	0.81 (3)	2.36 (3)	3.0442 (14)	143 (3)
O8—H8*A*⋯O3^i^	0.85 (3)	1.90 (3)	2.7497 (13)	171 (2)
O8—H8*B*⋯O1	0.92 (3)	1.77 (3)	2.6730 (14)	165 (3)
O9—H9*A*⋯O2^iii^	0.87 (3)	2.15 (3)	2.9472 (14)	152 (3)
O9—H9*B*⋯O5	0.79 (3)	1.96 (3)	2.7452 (14)	179 (3)

Symmetry codes: (i) 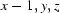; (ii) 
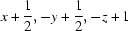
; (iii) 
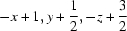
.

**Fig. 1.**
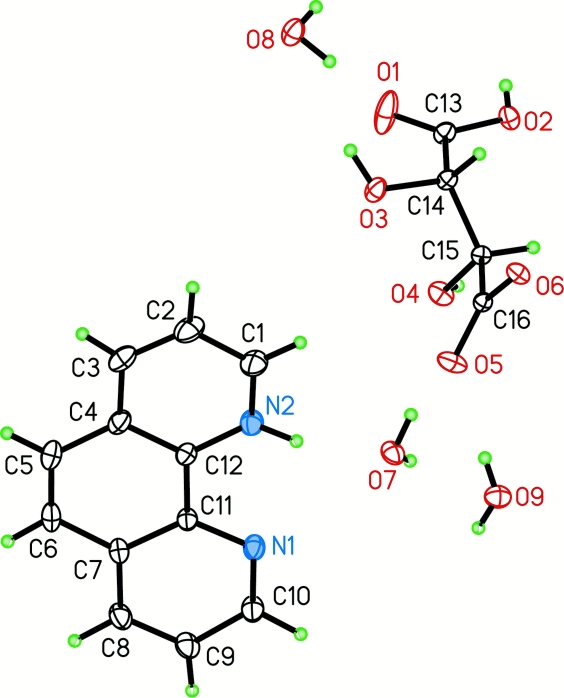
A drawing of the asymmetric unit of the title compound. Thermal ellipsoids are drawn at the 50% probability level.

**Fig. 2.**
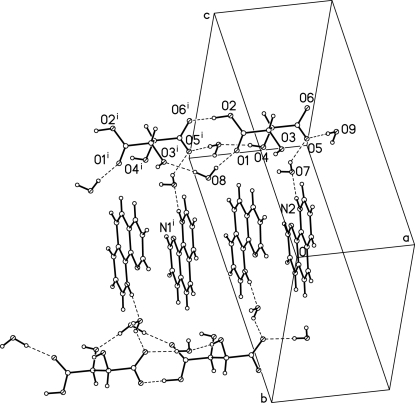
A view showing how the phenanthrolinium ions pack together in a parallel fashion while the tartrate anions string together via a strong hydrogen bond along the a direction of the crystal. Symmetry code: (i) x - 1, y, z.

**Fig. 3.**
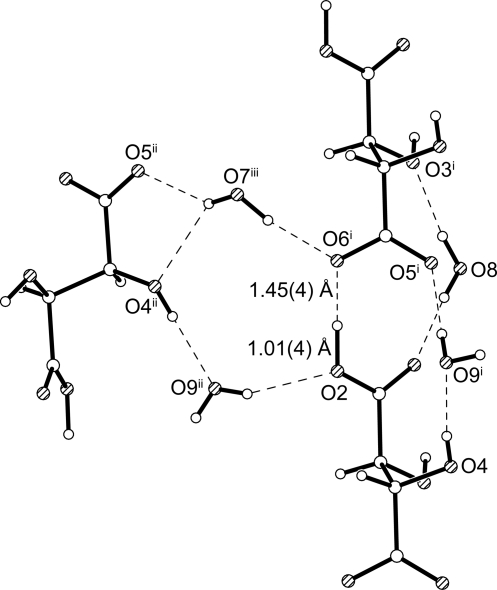
The hydrogen bonding interactions that support the strong hydrogen bonding interaction between tartrate anions. Symmetry codes: (i) x - 1, y, z; (ii) 1 - x, y - 1/2, 1.5 - z; (iii) -x, y - 1/2, 1.5 - z.

**Fig. 4.**
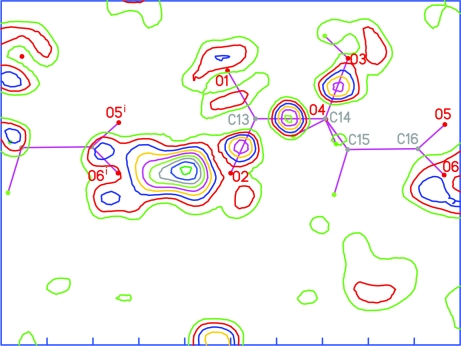
A plot of electron density contours showing the difference Fourier map for H2A, the hydrogen atom involved in the short hydrogen bond. The maximum density contour is 0.45 e/Å3.

## supplementary crystallographic information

### Comment

Tartaric acid is a colorless, diprotic organic acid that occurs naturally in many plants, particularly grapes, bananas, and tamarinds, and is one of the main acids found in wine. It is added to other foods to give a sour taste, and is used as an antioxidant. Many proton transfer compounds of tartaric acid and various bases have been reported, for example, (Paixão *et al.*, 1999; Bai *et al.*, 2005; Zhang *et al.*, 2006; Suresh *et al.*, 2006; Wang *et al.*, 2008; Su *et al.*, 2009). The title structure contains a cation of protonated 1,10-phenanthroline, an anion of mono-deprotonated D-tartaric acid, and three water molecules (Fig. 1). Thus, the crystal structure shows that one of the protons of the tartaric acid carboxylic groups has been transferred to one of the nitrogen atoms of the 1,10-phenanthroline molecule. A portion of the hydrogen bonding motif involving the anions, cations and water molecules is presented in Fig. 2; details of the O—H···O and N—H···O hydrogen bonds are given in Table 1. Fig. 2 also shows how the 1,10-phenanthrolinium rings are π-stacked such that they are perpendicular to the chain of tartrate anions that run along the *a* axis. The average perpendicular distance between the plane of N1/N2/C1/C2/C3/C4/C5/C6/C7/C8/C9/C10/C11/C12 and the 14 atoms at ii = 1/2 + *x*, 3/2 - *y*, 1 - *z* of the stacked phenanthroline ring is 3.58 (11) Å. The tartrate anions are connected head-to-tail by a short hydrogen bond between H2A, bonded to O2, and O6^i^ of the anion at i = *x* - 1, *y*, *z*. The O2—H2A distance is 1.01 (4) Å, H2A—O6^i^ is 1.45 (4) Å and O2···O6^i^ is 2.4554 (12) Å. The O2—H2A—O6^i^ angle is 174 (4)°. Electrostatic considerations, together with the use of resonance structures, could be used to explain the short hydrogen bond. Additionally, the existence of a number of supporting hydrogen bonds could be a factor, and these are depicted in Fig. 3. A similar head-to-tail arrangement with a short donor- acceptor distance is seen in some other hydrogen tartrate structures (Paixão *et al.*, 1999; Zhang *et al.*, 2006). The geometry of the hydrogen atom, H2A, that is involved in the short hydrogen bond has larger standard uncertainties than other hydrogen atoms in the structure. The larger uncertainty can be accounted for by examination of a plot of difference electron density (Fig. 4) (EDEN, Sheldrick (2008)), which shows that H2A resides in a shallow potential well that has a single minimum close to O2 but tails off towards O6^i^.

A previous structural determination of the isostructural *L*-tartrate enantiomer (II) (Wang *et al.*, 2006) missed the proton transfer and identified the compound as 1,10-phenanthroline (2*R*,3*R*)-tartaric acid. We have examined their data and confirmed that the proton transfer did occur, and refinement of the structure using the model of the title compound results in lower *R* values. Interestingly, a subsequent paper on the quinoline analog by Smith *et al.*, 2006, expressed surprise that the proton in (II) was not transferred: "···the absence of transfer in the *L*-tartaric acid-1,10-phenanthroline compound reported by Wang *et al.* (2006) when compared with the structurally similar [quinolinium] is not understood, considering that the p*K*_a_ value for 1,10-phenanthroline (4.86) is very close to that of quinoline (4.81)." We note that, in the structure of (II), the details of the short hydrogen bond are not revealed because tartaric acid O—H distance restraints of 0.82 (1) Å were applied, and also because the acceptor O atom has the misplaced H atom. In the refinement of the title compound, hydrogen atoms were freely refined. In (II), data were collected at 293 (2) K, and the data/parameter ratio is 7.34. In the title compound, data were collected at 90 (2) K, and the data/parameter ratio is 10.03.

### Experimental

The reaction between solutions of D-tartaric acid (7 mg, 1 mmol) in water (10 ml) and 1,10-phenanthroline (9 mg, 1 mmol) in methanol (5 ml) in a 1:1 molar ratio gave colorless rod crystals after slow evaporation of the solvent at room temperature.

### Refinement

Friedel opposites were merged. The absolute configuration followed from the use of D-tartaric acid as a starting material. Hydrogen atoms were located in a difference Fourier map and freely refined.

### Crystal data

**Table:**

C_12_H_9_N_2_^+^·C_4_H_5_O_6_^−^·3H_2_O	*F*(000) = 808
*M**_r_* = 384.34	*D*_x_ = 1.476 Mg m^−^^3^
Orthorhombic, *P*2_1_2_1_2_1_	Mo *K*α radiation, λ = 0.71073 Å
Hall symbol: P 2ac 2ab	Cell parameters from 9999 reflections
*a* = 7.1163 (14) Å	θ = 2.8–31.5°
*b* = 12.482 (3) Å	µ = 0.12 mm^−^^1^
*c* = 19.466 (4) Å	*T* = 90 K
*V* = 1729.2 (6) Å^3^	Rod, colourless
*Z* = 4	0.42 × 0.21 × 0.13 mm

### Data collection

**Table:**

Bruker SMART APEXII diffractometer	3251 independent reflections
Radiation source: fine-focus sealed tube	3149 reflections with *I* > 2σ(*I*)
graphite	*R*_int_ = 0.027
Detector resolution: 8.3 pixels mm^-1^	θ_max_ = 31.6°, θ_min_ = 1.9°
ω scans	*h* = −10→10
Absorption correction: multi-scan (*SADABS*; Sheldrick, 1996)	*k* = −18→18
*T*_min_ = 0.880, *T*_max_ = 0.984	*l* = −28→28
39094 measured reflections	

### Refinement

**Table:**

Refinement on *F*^2^	Primary atom site location: structure-invariant direct methods
Least-squares matrix: full	Secondary atom site location: difference Fourier map
*R*[*F*^2^ > 2σ(*F*^2^)] = 0.032	Hydrogen site location: difference Fourier map
*wR*(*F*^2^) = 0.089	All H-atom parameters refined
*S* = 1.06	*w* = 1/[σ^2^(*F*_o_^2^) + (0.0669*P*)^2^ + 0.1713*P*] where *P* = (*F*_o_^2^ + 2*F*_c_^2^)/3
3251 reflections	(Δ/σ)_max_ = 0.003
324 parameters	Δρ_max_ = 0.41 e Å^−^^3^
0 restraints	Δρ_min_ = −0.17 e Å^−^^3^

### Special details

**Table:**

Geometry. All e.s.d.'s (except the e.s.d. in the dihedral angle between two l.s. planes) are estimated using the full covariance matrix. The cell e.s.d.'s are taken into account individually in the estimation of e.s.d.'s in distances, angles and torsion angles; correlations between e.s.d.'s in cell parameters are only used when they are defined by crystal symmetry. An approximate (isotropic) treatment of cell e.s.d.'s is used for estimating e.s.d.'s involving l.s. planes.
Refinement. Refinement of *F*^2^ against ALL reflections. The weighted *R*-factor *wR* and goodness of fit *S* are based on *F*^2^, conventional *R*-factors *R* are based on *F*, with *F* set to zero for negative *F*^2^. The threshold expression of *F*^2^ > σ(*F*^2^) is used only for calculating *R*-factors(gt) *etc*. and is not relevant to the choice of reflections for refinement. *R*-factors based on *F*^2^ are statistically about twice as large as those based on *F*, and *R*- factors based on ALL data will be even larger.

### Fractional atomic coordinates and isotropic or equivalent isotropic displacement parameters (Å^2^)

**Table:**

	*x*	*y*	*z*	*U*_iso_*/*U*_eq_	
O1	−0.02434 (15)	0.28085 (14)	0.60625 (5)	0.0387 (3)	
O2	−0.01282 (11)	0.22520 (7)	0.71533 (4)	0.01530 (16)	
H2A	−0.155 (5)	0.227 (3)	0.7139 (19)	0.082 (11)*	
O3	0.34399 (12)	0.27724 (8)	0.59021 (4)	0.01825 (18)	
H3A	0.266 (3)	0.2529 (17)	0.5600 (11)	0.025 (5)*	
O4	0.29691 (13)	0.43589 (7)	0.69506 (5)	0.01723 (17)	
H4A	0.202 (4)	0.450 (2)	0.7164 (15)	0.052 (7)*	
O5	0.65945 (13)	0.40940 (7)	0.69497 (5)	0.02051 (18)	
O6	0.64269 (11)	0.23538 (7)	0.71823 (4)	0.01439 (15)	
N1	0.56039 (18)	0.85195 (8)	0.60703 (5)	0.01821 (19)	
N2	0.55402 (17)	0.64831 (8)	0.55452 (5)	0.01820 (19)	
H2B	0.558 (3)	0.6546 (15)	0.6011 (10)	0.020 (4)*	
C1	0.5526 (2)	0.54779 (10)	0.53258 (7)	0.0244 (3)	
H1	0.545 (4)	0.494 (2)	0.5654 (13)	0.038 (6)*	
C2	0.5479 (2)	0.52505 (11)	0.46232 (7)	0.0267 (3)	
H2	0.545 (4)	0.4546 (18)	0.4486 (12)	0.033 (5)*	
C3	0.5443 (2)	0.60764 (12)	0.41615 (7)	0.0238 (2)	
H3	0.533 (4)	0.590 (2)	0.3720 (13)	0.042 (6)*	
C4	0.54614 (19)	0.71407 (10)	0.43933 (6)	0.0194 (2)	
C5	0.5432 (2)	0.80340 (13)	0.39336 (6)	0.0263 (3)	
H5	0.533 (5)	0.792 (3)	0.3465 (16)	0.071 (9)*	
C6	0.5492 (2)	0.90440 (12)	0.41791 (6)	0.0266 (3)	
H6	0.549 (4)	0.966 (2)	0.3898 (14)	0.053 (8)*	
C7	0.55687 (19)	0.92492 (10)	0.49056 (6)	0.0190 (2)	
C8	0.5681 (2)	1.02891 (10)	0.51766 (7)	0.0217 (2)	
H8	0.568 (3)	1.0922 (17)	0.4873 (10)	0.026 (5)*	
C9	0.5760 (2)	1.04196 (10)	0.58747 (7)	0.0206 (2)	
H9	0.584 (3)	1.1120 (18)	0.6092 (10)	0.029 (5)*	
C10	0.5697 (2)	0.95135 (10)	0.63030 (6)	0.0201 (2)	
H10	0.577 (3)	0.9670 (17)	0.6820 (11)	0.027 (5)*	
C11	0.55581 (17)	0.83917 (9)	0.53766 (5)	0.01526 (19)	
C12	0.55133 (17)	0.73284 (9)	0.51062 (6)	0.0157 (2)	
C13	0.06195 (15)	0.25433 (10)	0.65712 (5)	0.01487 (19)	
C14	0.27564 (14)	0.25118 (9)	0.65602 (5)	0.01263 (18)	
H14	0.308 (3)	0.1803 (15)	0.6702 (9)	0.018 (4)*	
C15	0.35517 (14)	0.33050 (8)	0.70860 (5)	0.01159 (17)	
H15	0.316 (3)	0.3068 (14)	0.7552 (9)	0.017 (4)*	
C16	0.57000 (15)	0.32740 (8)	0.70659 (5)	0.01187 (18)	
O7	0.57458 (15)	0.62117 (7)	0.68894 (5)	0.01850 (17)	
H7A	0.491 (3)	0.6571 (19)	0.7160 (11)	0.034 (6)*	
H7B	0.549 (4)	0.558 (2)	0.6918 (12)	0.040 (6)*	
O8	−0.31974 (13)	0.30315 (9)	0.52265 (4)	0.02028 (18)	
H8A	−0.422 (4)	0.2882 (19)	0.5431 (12)	0.035 (6)*	
H8B	−0.231 (4)	0.301 (2)	0.5572 (12)	0.041 (6)*	
O9	0.97193 (13)	0.49415 (8)	0.75731 (5)	0.01928 (18)	
H9A	0.952 (4)	0.563 (2)	0.7552 (14)	0.047 (7)*	
H9B	0.883 (4)	0.4694 (19)	0.7395 (13)	0.038 (6)*	

### Atomic displacement parameters (Å^2^)

**Table:**

	*U*^11^	*U*^22^	*U*^33^	*U*^12^	*U*^13^	*U*^23^
O1	0.0136 (4)	0.0860 (11)	0.0166 (4)	0.0040 (5)	−0.0014 (3)	0.0110 (5)
O2	0.0102 (3)	0.0187 (4)	0.0171 (3)	0.0010 (3)	0.0016 (3)	0.0048 (3)
O3	0.0133 (3)	0.0298 (4)	0.0117 (3)	−0.0028 (3)	0.0021 (3)	−0.0019 (3)
O4	0.0153 (3)	0.0125 (3)	0.0240 (4)	0.0043 (3)	0.0014 (3)	0.0015 (3)
O5	0.0138 (3)	0.0135 (3)	0.0343 (5)	−0.0021 (3)	−0.0009 (3)	0.0033 (3)
O6	0.0097 (3)	0.0125 (3)	0.0209 (4)	0.0009 (3)	0.0011 (3)	0.0031 (3)
N1	0.0262 (5)	0.0151 (4)	0.0134 (4)	0.0032 (4)	0.0004 (4)	−0.0002 (3)
N2	0.0240 (5)	0.0154 (4)	0.0152 (4)	0.0024 (4)	−0.0009 (4)	−0.0013 (3)
C1	0.0332 (7)	0.0167 (5)	0.0234 (6)	0.0027 (5)	−0.0030 (5)	−0.0039 (4)
C2	0.0333 (7)	0.0210 (5)	0.0259 (6)	0.0034 (6)	−0.0050 (5)	−0.0095 (5)
C3	0.0255 (6)	0.0276 (6)	0.0182 (5)	0.0008 (5)	−0.0021 (5)	−0.0092 (4)
C4	0.0208 (5)	0.0238 (5)	0.0138 (4)	−0.0007 (5)	−0.0007 (4)	−0.0037 (4)
C5	0.0347 (7)	0.0318 (6)	0.0124 (4)	−0.0030 (6)	−0.0003 (5)	0.0008 (4)
C6	0.0376 (7)	0.0282 (6)	0.0140 (5)	−0.0036 (6)	−0.0008 (5)	0.0061 (4)
C7	0.0223 (5)	0.0198 (5)	0.0148 (4)	−0.0010 (5)	−0.0004 (4)	0.0041 (4)
C8	0.0266 (6)	0.0173 (5)	0.0214 (5)	0.0009 (5)	0.0001 (5)	0.0052 (4)
C9	0.0262 (5)	0.0139 (4)	0.0217 (5)	0.0034 (5)	0.0011 (5)	0.0016 (4)
C10	0.0292 (6)	0.0153 (4)	0.0158 (4)	0.0038 (5)	0.0011 (4)	−0.0006 (4)
C11	0.0173 (5)	0.0148 (4)	0.0137 (4)	0.0013 (4)	0.0003 (4)	0.0006 (3)
C12	0.0168 (5)	0.0168 (5)	0.0135 (4)	0.0006 (4)	−0.0005 (4)	−0.0012 (4)
C13	0.0097 (4)	0.0206 (5)	0.0143 (4)	−0.0004 (4)	0.0003 (3)	−0.0016 (4)
C14	0.0093 (4)	0.0161 (4)	0.0124 (4)	0.0003 (4)	0.0004 (3)	−0.0015 (3)
C15	0.0098 (4)	0.0116 (4)	0.0134 (4)	0.0011 (3)	−0.0002 (3)	−0.0001 (3)
C16	0.0105 (4)	0.0133 (4)	0.0118 (4)	0.0001 (4)	−0.0001 (3)	−0.0001 (3)
O7	0.0251 (4)	0.0114 (3)	0.0190 (4)	0.0008 (3)	0.0038 (3)	−0.0004 (3)
O8	0.0146 (4)	0.0316 (5)	0.0146 (3)	0.0005 (4)	0.0001 (3)	−0.0010 (3)
O9	0.0173 (4)	0.0166 (4)	0.0240 (4)	0.0006 (3)	−0.0010 (3)	−0.0015 (3)

### Geometric parameters (Å, °)

**Table:**

O1—C13	1.2113 (15)	C5—H5	0.92 (3)
O2—C13	1.3036 (13)	C6—C7	1.4384 (17)
O2—H2A	1.01 (4)	C6—H6	0.95 (3)
O3—C14	1.4082 (13)	C7—C8	1.4034 (18)
O3—H3A	0.86 (2)	C7—C11	1.4094 (16)
O4—C15	1.4043 (13)	C8—C9	1.3696 (18)
O4—H4A	0.81 (3)	C8—H8	0.99 (2)
O5—C16	1.2264 (14)	C9—C10	1.4059 (17)
O6—C16	1.2800 (13)	C9—H9	0.97 (2)
N1—C10	1.3226 (15)	C10—H10	1.03 (2)
N1—C11	1.3602 (14)	C11—C12	1.4282 (16)
N2—C1	1.3254 (16)	C13—C14	1.5214 (15)
N2—C12	1.3579 (15)	C14—C15	1.5324 (15)
N2—H2B	0.912 (19)	C14—H14	0.955 (19)
C1—C2	1.3973 (19)	C15—C16	1.5298 (15)
C1—H1	0.93 (3)	C15—H15	0.994 (17)
C2—C3	1.368 (2)	O7—H7A	0.91 (2)
C2—H2	0.92 (2)	O7—H7B	0.81 (3)
C3—C4	1.4030 (18)	O8—H8A	0.85 (3)
C3—H3	0.89 (3)	O8—H8B	0.92 (3)
C4—C12	1.4079 (15)	O9—H9A	0.87 (3)
C4—C5	1.4299 (19)	O9—H9B	0.79 (3)
C5—C6	1.349 (2)		
			
C13—O2—H2A	112 (2)	C8—C9—H9	122.7 (12)
C14—O3—H3A	108.6 (14)	C10—C9—H9	117.8 (12)
C15—O4—H4A	111 (2)	N1—C10—C9	123.59 (11)
C10—N1—C11	116.83 (10)	N1—C10—H10	121.1 (12)
C1—N2—C12	122.19 (11)	C9—C10—H10	115.2 (12)
C1—N2—H2B	113.7 (12)	N1—C11—C7	123.82 (11)
C12—N2—H2B	124.1 (12)	N1—C11—C12	118.39 (10)
N2—C1—C2	120.52 (12)	C7—C11—C12	117.79 (10)
N2—C1—H1	117.8 (15)	N2—C12—C4	119.43 (11)
C2—C1—H1	121.5 (15)	N2—C12—C11	119.33 (10)
C3—C2—C1	119.37 (12)	C4—C12—C11	121.23 (10)
C3—C2—H2	121.9 (14)	O1—C13—O2	125.45 (11)
C1—C2—H2	118.7 (14)	O1—C13—C14	120.15 (10)
C2—C3—C4	120.14 (11)	O2—C13—C14	114.40 (9)
C2—C3—H3	116.8 (17)	O3—C14—C13	110.62 (9)
C4—C3—H3	122.9 (17)	O3—C14—C15	109.32 (9)
C3—C4—C12	118.35 (11)	C13—C14—C15	110.06 (9)
C3—C4—C5	122.48 (11)	O3—C14—H14	113.3 (11)
C12—C4—C5	119.17 (12)	C13—C14—H14	105.1 (13)
C6—C5—C4	120.45 (11)	C15—C14—H14	108.4 (12)
C6—C5—H5	119 (2)	O4—C15—C16	108.30 (9)
C4—C5—H5	120 (2)	O4—C15—C14	111.77 (9)
C5—C6—C7	121.07 (12)	C16—C15—C14	109.61 (9)
C5—C6—H6	123.9 (16)	O4—C15—H15	111.5 (11)
C7—C6—H6	115.1 (16)	C16—C15—H15	107.4 (11)
C8—C7—C11	117.27 (11)	C14—C15—H15	108.2 (11)
C8—C7—C6	122.45 (11)	O5—C16—O6	124.88 (10)
C11—C7—C6	120.27 (12)	O5—C16—C15	120.16 (10)
C9—C8—C7	119.03 (11)	O6—C16—C15	114.96 (9)
C9—C8—H8	120.0 (12)	H7A—O7—H7B	107 (2)
C7—C8—H8	121.0 (12)	H8A—O8—H8B	104 (2)
C8—C9—C10	119.43 (11)	H9A—O9—H9B	104 (3)
			
C12—N2—C1—C2	−0.1 (2)	C1—N2—C12—C11	−179.10 (13)
N2—C1—C2—C3	−0.2 (3)	C3—C4—C12—N2	−0.10 (19)
C1—C2—C3—C4	0.3 (2)	C5—C4—C12—N2	179.96 (12)
C2—C3—C4—C12	−0.2 (2)	C3—C4—C12—C11	179.23 (12)
C2—C3—C4—C5	179.78 (15)	C5—C4—C12—C11	−0.71 (19)
C3—C4—C5—C6	−178.60 (15)	N1—C11—C12—N2	−0.78 (18)
C12—C4—C5—C6	1.3 (2)	C7—C11—C12—N2	178.68 (12)
C4—C5—C6—C7	−0.6 (3)	N1—C11—C12—C4	179.89 (12)
C5—C6—C7—C8	178.30 (15)	C7—C11—C12—C4	−0.65 (18)
C5—C6—C7—C11	−0.8 (2)	O1—C13—C14—O3	−2.66 (18)
C11—C7—C8—C9	−0.5 (2)	O2—C13—C14—O3	176.32 (9)
C6—C7—C8—C9	−179.65 (14)	O1—C13—C14—C15	118.25 (14)
C7—C8—C9—C10	−1.0 (2)	O2—C13—C14—C15	−62.78 (13)
C11—N1—C10—C9	−0.3 (2)	O3—C14—C15—O4	62.27 (11)
C8—C9—C10—N1	1.5 (2)	C13—C14—C15—O4	−59.41 (12)
C10—N1—C11—C7	−1.2 (2)	O3—C14—C15—C16	−57.81 (11)
C10—N1—C11—C12	178.18 (12)	C13—C14—C15—C16	−179.49 (9)
C8—C7—C11—N1	1.7 (2)	O4—C15—C16—O5	0.45 (14)
C6—C7—C11—N1	−179.15 (14)	C14—C15—C16—O5	122.63 (11)
C8—C7—C11—C12	−177.76 (12)	O4—C15—C16—O6	179.93 (9)
C6—C7—C11—C12	1.42 (19)	C14—C15—C16—O6	−57.89 (12)
C1—N2—C12—C4	0.2 (2)		

### Hydrogen-bond geometry (Å, °)

**Table:**

*D*—H···*A*	*D*—H	H···*A*	*D*···*A*	*D*—H···*A*
O2—H2A···O6^i^	1.01 (4)	1.45 (4)	2.4554 (12)	174 (4)
O3—H3A···O8^ii^	0.86 (2)	1.86 (2)	2.6817 (13)	159 (2)
O4—H4A···O9^i^	0.81 (3)	1.90 (3)	2.7102 (13)	173 (3)
N2—H2B···O7	0.912 (19)	1.763 (19)	2.6426 (14)	161.3 (18)
O7—H7A···O6^iii^	0.91 (2)	1.87 (2)	2.7727 (13)	170 (2)
O7—H7B···O5	0.81 (3)	2.02 (3)	2.7141 (14)	144 (3)
O7—H7B···O4	0.81 (3)	2.36 (3)	3.0442 (14)	143 (3)
O8—H8A···O3^i^	0.85 (3)	1.90 (3)	2.7497 (13)	171 (2)
O8—H8B···O1	0.92 (3)	1.77 (3)	2.6730 (14)	165 (3)
O9—H9A···O2^iii^	0.87 (3)	2.15 (3)	2.9472 (14)	152 (3)
O9—H9B···O5	0.79 (3)	1.96 (3)	2.7452 (14)	179 (3)

Symmetry codes: (i) *x*−1, *y*, *z*; (ii) *x*+1/2, −*y*+1/2, −*z*+1; (iii) −*x*+1, *y*+1/2, −*z*+3/2.
